# Toward microbiome-assisted remediation: Vanadium–titanium magnetite mining reshapes cropland soil chemistry and rhizosphere microbiomes

**DOI:** 10.1007/s44297-026-00072-9

**Published:** 2026-04-02

**Authors:** Bingliang Liu, Xiao Huang, Cheng Chang, Xin Wan, Mingrong Liu, Rui Li, Jun Li, Qiang Li, Yang Tao

**Affiliations:** 1 College of Food and Biological Engineering, Chengdu University, Chengdu, China; 2 Sichuan-Xizang Medicinal Resource Breeding and Standardization Team, Institute for Advanced Study, Chengdu University, Chengdu, China; 3 School of Vanadium and Titanium, Panzhihua University, Panzhihua, China

**Keywords:** Crop health, VTM mining, Heavy-metal stress, Rhizosphere microbiome, Microbiome-assisted remediation

## Abstract

Vanadium–titanium magnetite (VTM) mining can modify cropland soils and root-associated microbiomes with implications for crop health. However, how crop rhizosphere microbiomes reorganize under VTM-derived stress—particularly when bacteria and fungi are considered together—remains poorly understood. In the Hongge district (Panzhihua, China), we sampled bulk soils and rhizospheres of lettuce, rapeseed, and pea from croplands within the VTM mining influence zone (mining-impacted fields) and paired croplands outside the zone (reference fields). We measured soil chemistry and profiled bacterial and fungal communities using 16S rRNA and ITS amplicon sequencing, respectively. Mining-impacted soils generally showed a VTM geochemical imprint, neutral–alkaline pH, and reduced plant-available P and K. Bray–Curtis–based ordinations indicated a clear separation between mining-impacted and reference rhizospheres, and taxonomic profiles suggested host-dependent reassembly of both bacterial and fungal communities. Putative functional profiling suggested a shift toward stress-accommodation processes, and fungal guild assignments tended to tilt toward saprotrophic/endophytic categories. Mantel analyses identified pH as one of the strongest correlates of community turnover, whereas structural equation modeling was consistent with nutrient availability (available N/P/K composite) explaining a substantial portion of the VTM effect; the composite total-metal-load axis (Fe/V/Ti/Zn) showed limited explanatory power, especially for fungi. Together, these field-based, two-kingdom signals link VTM-altered soil chemistry to rhizosphere restructuring in edible crops and provide actionable indicators for crop health management.

**Supplementary Information:**

The online version contains supplementary material available at 10.1007/s44297-026-00072-9.

## Introduction

Mining and smelting can introduce excessive heavy metals into surrounding environments, posing documented risks to soil ecosystems and, indirectly, to food safety [1]. The Panzhihua region of Sichuan Province, Southwest China, is renowned for its rich vanadium–titanium magnetite (VTM) resources; the nearby Hongge VTM mine is among China’s largest V–Ti–Fe oxide deposits and ranks near the top nationally in vanadium reserves [2–4]. While VTM mining is of great economic importance, the extraction and processing activities have led to the release of vanadium, titanium, iron, and co-occurring metals into surrounding soils through ore dust, wastewater, and slag deposition [5]. Consequently, agricultural lands adjacent to mining areas have experienced notable heavy metal accumulation and degradation of soil ecological function.

Vanadium (V) is an emerging contaminant of concern due to its persistence and toxicity in the environment [6]. Soils in the Panzhihua VTM mining area have been reported to contain vanadium concentrations ranging from approximately 150 mg·kg^−1^ up to nearly 4800 mg·kg^−1^, far exceeding the natural background level (~ 82 mg·kg^−1^) [7]. Elevated vanadium and associated metals (e.g., Ti, Fe, Zn) in soil can harm plant growth and soil biota. Heavy metal stress can disrupt soil microbial processes such as nutrient cycling and organic matter decomposition, ultimately impairing soil fertility and health [8]. Moreover, high levels of vanadium and other metals have been shown to significantly alter soil microbial community structure, favoring metal-tolerant populations while reducing overall diversity [9, 10]. For instance, Cao et al. [7] found that prolonged V contamination in Panzhihua soils led to a proliferation of heavy metal–resistant bacteria and fungi, accompanied by a decline in less-tolerant species. Such edaphic shifts threaten crop nutrient acquisition, stress resilience, and disease suppression—core dimensions of crop health.

The rhizosphere—the narrow soil zone influenced by roots—hosts densely interactive microbial communities that underpin plant nutrition, stress tolerance, and disease suppression [11–13]. In addition to diverse fungal guilds (mutualists such as arbuscular mycorrhizal fungi, saprotrophs, and pathogens), bacteria are pivotal: plant growth–promoting rhizobacteria mobilize nutrients (N, P, Fe via nitrification, phosphatases, and siderophores), produce phytohormones, form root-associated biofilms, and detoxify metals through sequestration, efflux, and redox transformations [14, 15]. Heavy-metal inputs can perturb this balance in both kingdoms but not uniformly across guilds. Beneficial mycorrhizae are often metal-sensitive and may be suppressed at high loads [16], whereas certain opportunistic or metal-tolerant fungi (including some saprotrophs and pathogens) can persist or even proliferate in contaminated soils [7, 17]. Similarly, rhizosphere bacterial assemblages frequently exhibit shifts in evenness and composition [18–20]. Recent work across mining- and smelter-impacted agroecosystems indicates that heavy-metal contamination can reshape rhizosphere microbiomes by altering diversity, taxonomic composition, and functional potential, often selecting for stress-tolerant lineages while disfavoring metal-sensitive groups [21–23]. These responses are frequently context dependent and can vary with host identity, contamination history, and the co-varying edaphic template (e.g., pH, organic matter, and nutrient pools) that modulates metal bioavailability and microbial niche availability. Network-based analyses further suggest that metal contamination can reorganize microbial co-occurrence patterns and may shift “keystone” roles toward broadly distributed generalists [24], although the direction and magnitude of network changes can differ among systems. Collectively, these findings support a view in which heavy metals act together with pH–nutrient regimes to filter rhizosphere communities, motivating integrated, multi-kingdom field assessments that link soil chemistry to both bacterial and fungal turnover.

In the VTM context of Panzhihua, existing work has provided important insights from bulk soils and from targeted microbial groups (e.g., selected bacterial indicators; [7]). However, how crop rhizosphere microbiomes reorganize under VTM-derived stress—especially when considering bacteria and fungi together—remains less well resolved. This gap matters because plant-mediated, two-kingdom shifts can propagate to nutrient cycling, crop performance, and agroecosystem resilience under contamination. Accordingly, we addressed two overarching questions: (i) How does VTM mining reshape the soil template in adjacent croplands (metals, pH, and nutrient availability) and, in turn, the composition of bacterial and fungal rhizosphere communities across contrasting crop hosts? (ii) Among the co-varying edaphic factors, which pathways most plausibly underpin community turnover?

To answer these questions, we conducted a field study around the Hongge VTM district, sampling paired croplands within the mining influence zone and outside the zone. We collected bulk soils (geochemical context) and rhizospheres from lettuce, rapeseed, and pea; quantified key soil properties and total metals (Fe, V, Ti, Zn); and profiled bacterial (16S rRNA) and fungal (ITS) communities. We further identified indicator clades (LEfSe), inferred putative functional/guild shifts (PICRUSt2; FUNGuild), and linked community turnover to environmental gradients using β-diversity analyses, genus–environment correlations, and Mantel tests. Finally, we applied structural equation modeling (SEM) to partition direct and indirect pathways from VTM exposure to community shifts. Together, this two-kingdom, rhizosphere-focused framework provides an integrated view of how VTM-altered soil chemistry reshapes crop-associated microbiomes and helps prioritize microbial and edaphic indicators for management in metal-impacted farmlands.

## Results

### Soil physicochemical properties and heavy metal concentrations

Key soil properties and total metal contents are summarized in Table S1. Soils were neutral to slightly alkaline (pH 7.01–7.97), with the highest pH in mining-affected bulk soil (VT-CK, 7.97) compared to the reference bulk soil (CK, 7.05). Among rhizospheres, pea at the mining site (VT-Psa) showed notably high pH (7.88), while lettuce rhizosphere pH decreased relative to its control (7.36 vs. 7.80).

Soil organic carbon (OC) and total macronutrients (N, P, K) were generally lower at the mining site. OC declined most sharply in mining-affected lettuce rhizosphere (− 64.1%), while available phosphorus (AP) and potassium (AK) were severely depleted—mining rhizospheres retained only 8–39% of AP and 18–46% of AK relative to clean rhizospheres.

Heavy metal enrichment was crop-dependent. The pea rhizosphere at the mining site (VT-Psa) exhibited the strongest accumulation, with Fe, V, and Ti levels approximately 2.0-, 2.3-, and 2.5-fold higher than those in the clean pea rhizosphere. In contrast, metal increases were moderate in lettuce, and rapeseed even showed lower metal contents in the mining rhizosphere compared to its control. Zinc was highest in lettuce rhizospheres at both sites (∼100–108 mg·kg⁻^1^).

In summary, mining‐affected soils are characterized by depleted OC and macronutrients, severe reductions in available P and K, and marked but crop-dependent metal enrichment—most pronounced in the pea rhizosphere at the VTM site. These chemical shifts set the context for subsequent analyses of rhizosphere microbial responses.

### Alpha diversity of rhizosphere bacteria and fungi

Across 24 rhizosphere samples (3 replicates × 8 groups; 6 rhizospheres + 2 bulk soils), we obtained 1,611,224 high-quality 16S rRNA gene reads and 1,840,701 high-quality ITS tags (Data S1), clustering into 17,963 bacterial ASVs (Data S2) and 2,245 fungal ASVs (Data S3).

**Fig. 1:**
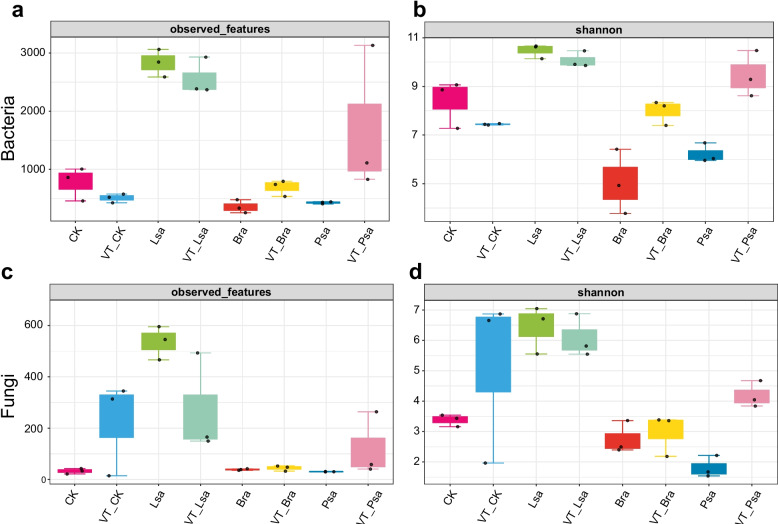
Alpha diversity of rhizosphere microbiomes. Boxplots show observed ASVs (richness) (**a**, **c**) and Shannon diversity (**b**, **d**) for bacterial (16S) and fungal (ITS) communities across soil type × crop groups. Points represent biological replicates (*n* = 3 per group). Statistical comparisons were performed within each crop rhizosphere (VTM vs. reference), while bulk-soil controls (CK, VT-CK) were evaluated separately and not pooled with rhizosphere samples. Lsa, *Lactuca sativa*; Bra, *Brassica rapa*; Psa, *Pisum sativum*

Alpha‐diversity patterns (Fig. 1; Fig. S2) were crop dependent. Lettuce (Lsa vs VT-Lsa) showed modest bacterial declines (observed 2829.0 → 2560.7, − 9.5%; Chao1 3101.3 → 2769.8, − 10.7%; Shannon 10.47 → 10.08, − 3.8%), with slight drops in Simpson and Pielou, whereas fungal richness contracted sharply (observed 535.3 → 269.7, ≈ − 50%; Chao1 575.6 → 272.1, ≈ − 53%), with a small Shannon decrease (6.44 → 6.08), minor Simpson decline, and higher evenness (Pielou 0.710 → 0.775). Rapeseed (Bra vs VT-Bra) exhibited pronounced bacterial increases (observed 355.7 → 691.0, + 94.3%; Chao1 393.4 → 721.5, + 83.4%; Shannon 5.04 → 7.98, + 58.3%), accompanied by higher Simpson and Pielou (0.594 → 0.847), while fungal metrics were broadly similar to slightly higher at the VTM site (observed 39.0 → 44.7; Chao1 42.2 → 45.5; Shannon 2.75 → 2.97; modest Simpson rise; Pielou 0.519 → 0.541). Pea (Psa vs VT-Psa) showed the strongest bacterial gains (observed 428.7 → 1691.0, + 295%; Chao1 467.9 → 1839.5, + 293%; Shannon 6.22 → 9.46, + 52%), with higher Simpson and Pielou (0.712 → 0.903) and a parallel fungal increase (observed 30.7 → 121.3; Chao1 31.96 → 123.86; Shannon 1.81 → 4.19; Simpson up; Pielou 0.366 → 0.663). Overall, α-diversity responses were heterogeneous and crop-dependent: lettuce declined (especially fungal richness), rapeseed increased mainly in bacteria, and pea increased in both kingdoms at the VTM site.

### Beta diversity and community structure of bacteria and fungi

**Fig. 2:**
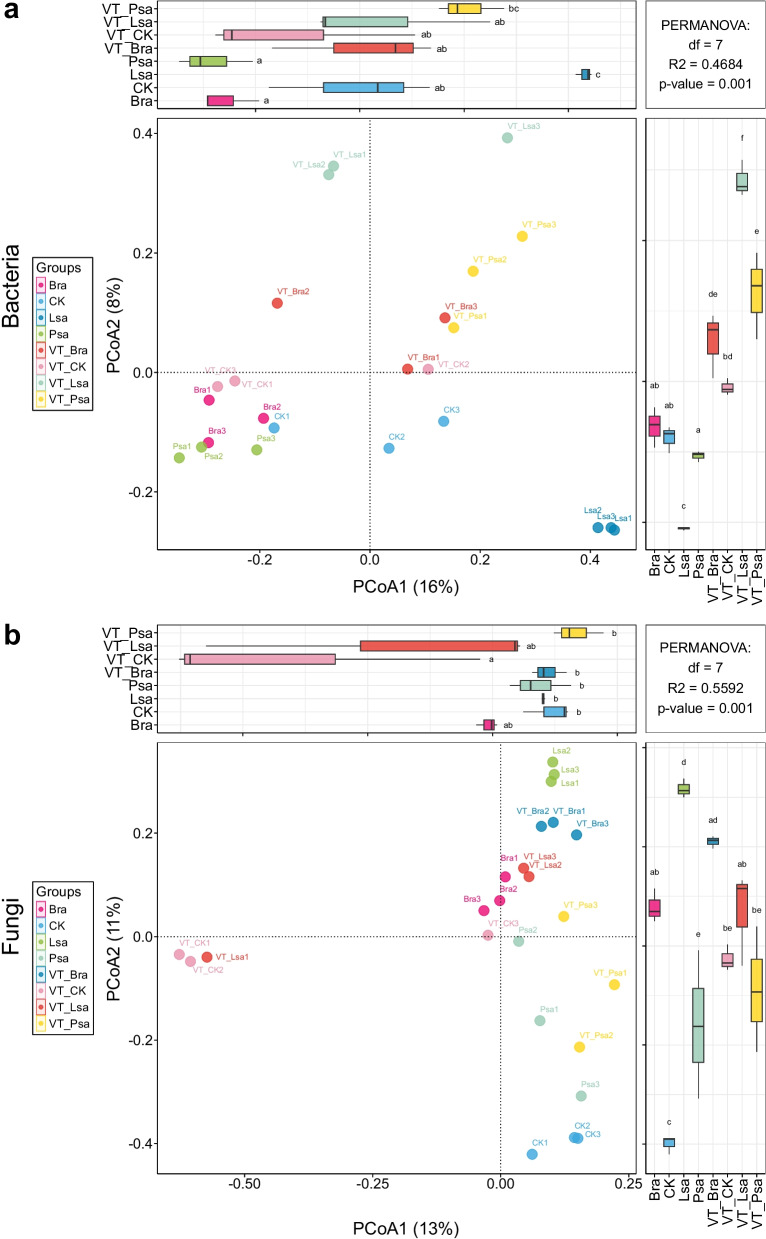
Beta diversity of rhizosphere microbiomes. **a** Bacteria; **b** Fungi. Ordinations are based on Bray–Curtis dissimilarities using principal coordinates analysis (PCoA) (axis labels show % variance explained). Points are biological replicates; colors/shapes denote treatment groups (CK, VT-CK, Lsa, VT-Lsa, Bra, VT-Bra, Psa, VT-Psa). The top and right-side boxplots summarize group distributions of the corresponding axis scores; gray insets report PERMANOVA results (vegan adonis2: R^2^ and *p*-value). Lsa, *Lactuca sativa*; Bra, *Brassica rapa*; Psa, *Pisum sativum*

Beta diversity (Bray–Curtis) revealed clear, host-dependent restructuring of rhizosphere microbiomes at the VTM site (Fig. 2). For bacteria (Fig. 2a), principal coordinates analysis (PCoA) separated VTM from reference rhizospheres along the primary axis (PCoA1 = 16%; PCoA2 = 8%), with additional crop-wise sub-clustering; among VTM groups, VT-Psa showed the largest displacement, and VTM clusters tended to be more dispersed, consistent with greater between-sample heterogeneity under metal stress. Non-metric multidimensional scaling (NMDS) recovered the same grouping pattern (Fig. S3a). Permutational multivariate analysis of variance (PERMANOVA) on Bray–Curtis distances confirmed strong among-group compositional differences. For fungi (Fig. 2b), PCoA likewise separated VTM from reference rhizospheres (PCoA1 = 13%; PCoA2 = 11%) and further resolved crop-specific sub-clusters; VT-Psa again shifted most relative to its control, indicating pronounced remodeling of its fungal assemblage, and VTM fungal points were also more disperse. NMDS corroborated the PCoA structure (Fig. S3b), and PERMANOVA indicated significant differences among groups.

To explicitly test whether the mining effect differed among crop rhizospheres, we conducted a two-factor PERMANOVA on Bray–Curtis dissimilarities for rhizosphere samples only (factors: Crop, VTM exposure, and their interaction) (Tables S2–S3). For bacteria, both Crop (R^2^ = 0.178, *F* = 2.089, *P* = 0.001) and VTM exposure (R^2^ = 0.114, *F* = 2.671, *P* = 0.002) were significant, and a significant Crop × VTM interaction was also detected (R^2^ = 0.197, *F* = 2.314, *P* = 0.001), indicating crop-dependent shifts in bacterial community composition under VTM conditions. For fungi, Crop explained the largest fraction of compositional variance (R^2^ = 0.240, *F* = 2.37, *P* = 0.001), and both VTM exposure (R^2^ = 0.106, *F* = 2.27, *P* = 0.001) and the Crop × VTM interaction (R^2^ = 0.189, *F* = 2.44, *P* = 0.001) were significant, supporting crop-specific fungal responses to mining-associated conditions.

### Taxonomic composition of rhizosphere bacterial and fungal communities

**Fig. 3:**
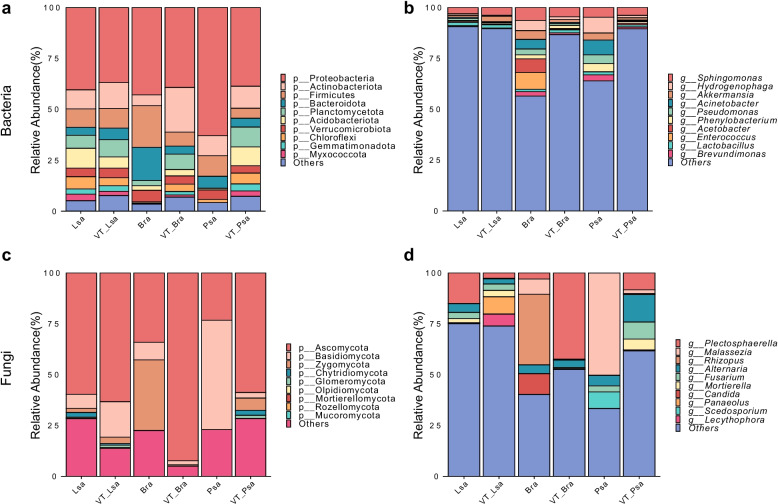
Taxonomic composition of rhizosphere microbiomes. Bacterial panels: **a** phylum level (top 10), **b** genus level (top 10); fungal panels: **c** phylum level (top 9), **d** genus level (top 10). Stacked bars show relative abundance (%) per sample and treatment group (Lsa, VT_Lsa, Bra, VT_Bra, Psa, VT_Psa); low-abundance taxa are pooled as “Others.” Unclassified reads/taxa were removed prior to plotting, and proportions were re-normalized to 100%. Colors denote taxa; bars sum to 100%. Rank prefixes in legends follow common microbiome conventions (e.g., p__ for phylum, g__ for genus). Lsa, *Lactuca sativa*; Bra, *Brassica rapa*; Psa, *Pisum sativum*

At the phylum level, rhizosphere bacterial communities across the three crops were consistently dominated by Proteobacteria, accompanied to varying degrees by Actinobacteriota, Firmicutes, and Bacteroidota; lettuce and pea showed Proteobacteria-skewed profiles at both sites, whereas rapeseed was more even, with Myxococcota, Actinobacteriota, Planctomycetota, and Chloroflexi relatively elevated in VT-Bra (Fig. 3a). This pattern is consistent with the general observation that carbon-rich rhizospheres enrich copiotrophs (e.g., Proteobacteria, Bacteroidota), while oligotrophic lineages are comparatively reduced [25], reflecting selection by fast-cycling, C-replete root zones. Genus-level (top 10) changes were crop-specific (Fig. 3b). In lettuce (VT-Lsa), *Sphingomonas*, *Akkermansia*, and *Phenylobacterium* increased relative to the reference. In contrast, rapeseed (VT-Bra) showed reductions in *Enterococcus*, *Brevundimonas*, *Acetobacter*, *Hydrogenophaga*, *Acinetobacter*, and *Pseudomonas*. Pea rhizospheres (VT-Psa) were characterized by decreases in *Pseudomonas*, *Hydrogenophaga*, *Akkermansia*, *Acinetobacter*, *Phenylobacterium*, and *Brevundimonas*, alongside an increase in *Acetobacter*. *Sphingomonas* is a typical rhizosphere colonizer with plant growth-promoting and stress-tolerance potential (including reported heavy-metal tolerance/adsorption) [26], which could contribute to its persistence or expansion under stress.

For fungi, all samples were overwhelmingly dominated by Ascomycota at the phylum level, followed by Basidiomycota, with smaller fractions from Chytridiomycota and Glomeromycota (Fig. 3c). Such Ascomycota predominance is widely documented in agricultural soils and is reproduced here at both sites [27, 28]. Fungal genus-level (top 10) turnover was pronounced and host-dependent (Fig. 3d). In lettuce, *Plectosphaerella* decreased, while *Panaeolus* and *Lecythophora* increased. The rapeseed rhizosphere was marked by an increase in *Plectosphaerella* and *Mortierella*, concurrent with decreases in *Rhizopus*, *Malassezia*, and *Candida*. The most complex shift occurred in pea, which showed increases in *Plectosphaerella*, *Alternaria*, *Fusarium*, *Mortierella*, *Lecythophora*, and *Rhizopus* but decreases in *Malassezia* and *Scedosporium*. *Mortierella* is a common soil–rhizosphere saprotroph, with many strains reported as plant growth–promoting fungi [29]; its increase in VT-Bra might be associated with stress-altered rhizosphere carbon and ionic conditions. Conversely, several genera common at the reference site declined at the mining site, indicating community reassembly and turnover under metal stress—consistent with evidence that heavy metal pollution reshapes soil microbial composition [30].

### Microbial biomarkers and co-occurrence patterns under VTM exposure

**Fig. 4:**
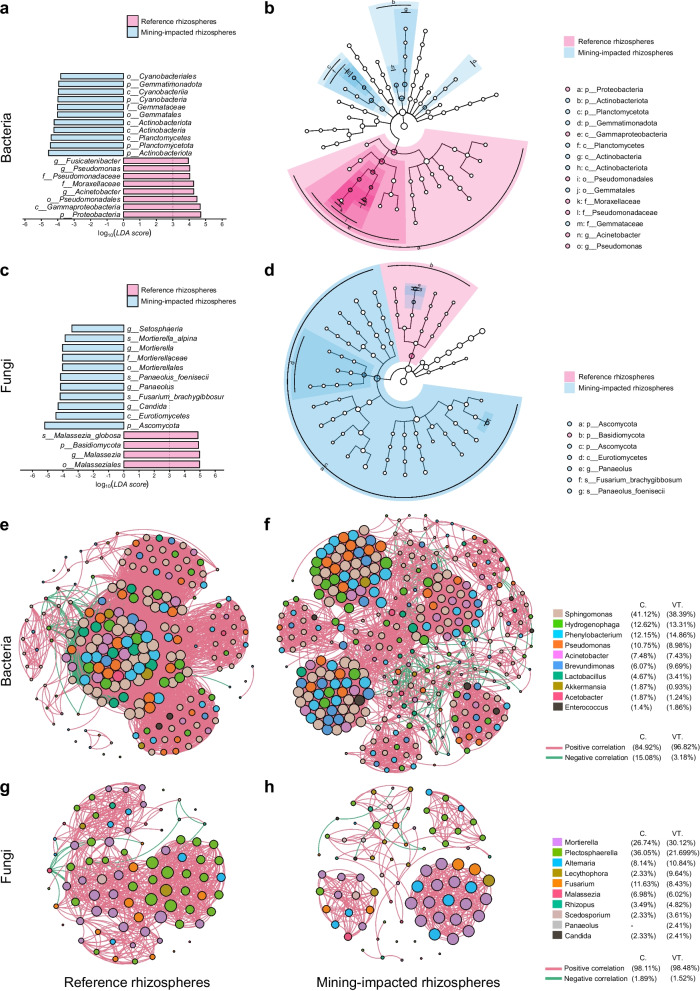
LEfSe biomarkers and co-occurrence networks of rhizosphere microbiota in response to VTM exposure. Rhizosphere samples from the three crops were pooled by status (pink, reference rhizospheres; blue, mining-impacted rhizospheres); bulk soils (CK, VT-CK) were excluded to avoid niche confounding. **a** Bacteria—differential taxa and their effect sizes (log10 LDA scores). **b** Bacteria-cladogram showing the phylogenetic distribution of discriminant clades; colored sectors indicate enrichment in the corresponding group. **c** Fungi—differential taxa and LDA scores. **d** Fungi-cladogram of discriminant fungal clades. Only taxa meeting the preset LEfSe significance/LDA thresholds are displayed; rank prefixes follow standard notation (p_, c_, o_, f_, g_, s_). **e**–**h** Co‑occurrence networks of dominant bacterial and fungal genera in reference and VTM‑exposed rhizospheres. Networks were inferred from genus-level relative abundances using significant Spearman correlations. Node color indicates taxon identity (legend), node size reflects mean relative abundance, and edge color represents positive (pink) or negative (green) correlations

To capture the overall impact of VTM exposure on crop rhizospheres, we pooled all planted rhizosphere samples by contamination status—mining-impacted rhizospheres (within the VTM influence zone) versus reference rhizospheres (paired croplands outside the zone)—and excluded bulk soils (CK, VT-CK) to avoid niche confounding. Using LEfSe—which couples nonparametric tests with linear discriminant analysis to identify clade-consistent biomarkers and quantify their effect sizes [31]—we detected clear, kingdom-specific shifts between the two groups (Fig. 4; Data S4). For bacteria, mining-impacted rhizospheres were enriched in several higher-rank clades typical of stress-tolerant/oligotrophic guilds, including Actinobacteriota/Actinobacteria, Gemmatimonadota/Gemmatales, Cyanobacteria/Cyanobacteriales, and Planctomycetota/Planctomycetes (blue in Fig. 4a, b). In contrast, reference rhizospheres were characterized by a Proteobacteria lineage (Gammaproteobacteria → Pseudomonadales → Pseudomonadaceae/Moraxellaceae) with prominent indicator genera *Pseudomonas* and *Acinetobacter*, and *Fusicatenibacter* also discriminated the reference rhizospheres (pink in Fig. 4a, b). These results could suggest a shift toward resource-limited or physicochemically challenging conditions, whereas Proteobacteria-dominated assemblages—especially Pseudomonadales—remain relatively more prevalent in the reference fields; notably, members of *Pseudomonas* are well documented for rhizosphere competence and heavy-metal tolerance [32, 33], consistent with their prominence in our LEfSe output.

In fungal communities, mining-impacted fields were enriched primarily within Ascomycota—indicator taxa included *Candida*, *Fusarium* (e.g., *F. brachygibbosum*), and *Setosphaeria*—with additional gains in Mortierellomycota (*Mortierella*, e.g., *M. alpina*) and Basidiomycota (*Panaeolus* s.l., e.g., *P. foenisecii*) (blue, Fig. 4c, d). In contrast, reference rhizospheres were characterized by Basidiomycota, especially Malasseziales/*Malassezia* (e.g., *M. globosa*) (pink, Fig. 4c, d). The prominence of *Mortierella* at VT sites is consistent with reported plant-growth-promoting and stress-adaptive traits [29]. Together, these biomarkers may provide a concise panel for crop-health monitoring in VTM-impacted fields.

Next, we conducted genus-level co-occurrence network analysis for bacterial and fungal communities in the pooled reference rhizospheres and mining-impacted rhizospheres (Fig. 4e–h). For bacteria, both networks were characterized predominantly by positive associations, but the VTM network exhibited a higher proportion of positive links (96.82%) than the reference network (84.92%), accompanied by a relative reduction in negative correlations (Fig. 4e, f). Topological indices further indicated that, relative to the reference network, the VTM bacterial network was less densely connected (lower density and average degree and longer average path length) yet more compartmentalized (higher modularity), consistent with a re-arranged modular architecture under VTM-associated conditions (Table S4). Core genera such as *Sphingomonas*, *Hydrogenophaga*, and *Phenylobacterium* appeared as central nodes in both contexts, consistent with their prominence in the LEfSe results. However, the VTM network also showed distinct clusters and altered connectivity patterns, which might reflect shifts in potential ecological niches or stress-associated co-occurrence under altered soil chemistry [34]. Fungal networks likewise displayed predominantly positive correlations in both groups (Fig. 4g, h). In contrast to the reference network, the VTM fungal network showed reduced overall connectivity but higher modularity and clustering coefficients, indicating a more module-structured association architecture under VTM exposure (Table S4). *Mortierella* and *Alternaria* were prominent in both networks, in line with their differential abundance signals; VTM exposure was also associated with module-specific rewiring involving opportunistic/tolerant genera such as *Lecythophora* and *Scedosporium*. Overall, the LEfSe results and these co-occurrence patterns support that VTM exposure is accompanied by changes in the way dominant bacterial and fungal genera are interrelated, potentially reflecting a broad reorganization of putative associations in response to adaptive shifts in soil conditions.

### Functional guild analysis of rhizosphere microbiomes (STAMP)

**Fig. 5:**
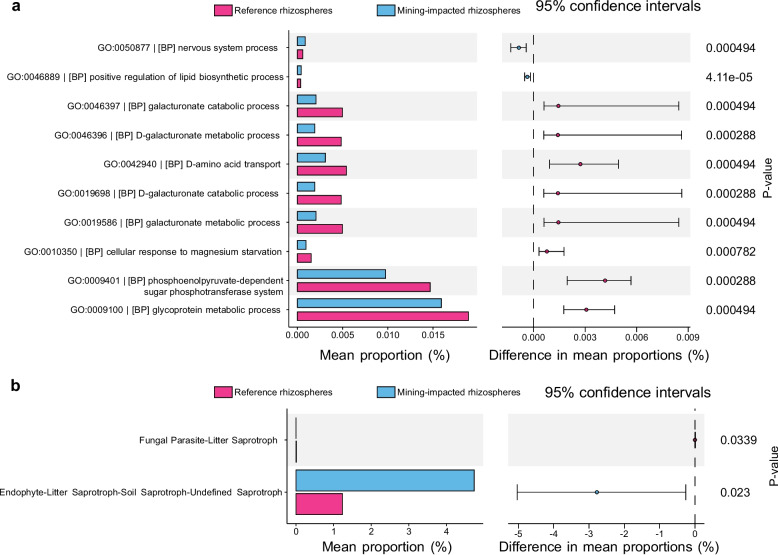
Bacterial functional profiles and fungal guilds of rhizosphere microbiomes (STAMP). **a** Bacteria: PICRUSt2-inferred GO Biological Process terms showing the top differentially abundant functions between pooled groups (pink, reference rhizospheres; blue, mining-impacted rhizospheres). Left bars give mean proportions (%); right forest plots show between-group differences with 95% CIs (Wilcox’s tests; *P* < 0.05). **b** Fungi: FUNGuild trophic modes with significant between-group differences (*P* < 0.05); bars and forest plots are displayed as in panel (**a**). Functional predictions were generated with PICRUSt2 and fungal guilds assigned with FUNGuild; statistics and visualization were performed in STAMP

Pooling planted rhizosphere samples by contamination status (mining-impacted rhizospheres vs. reference rhizospheres) and testing in STAMP, we observed coherent, kingdom-specific functional shifts (Fig. 5). For bacteria (Fig. 5a), PICRUSt2-inferred GO Biological Process terms (*P* < 0.05) indicated that carbohydrate utilization modules were relatively higher in the reference fields, including glycoprotein metabolic process and the phosphoenolpyruvate-dependent sugar phosphotransferase system, alongside several galacturonate catabolic/metabolic processes; in contrast, stress- and membrane-related functions tended to be higher at the VTM site, including cellular response to magnesium starvation, D-amino-acid transport, and positive regulation of lipid biosynthetic process. This apparent trade-off—from substrate acquisition toward membrane/ion-homeostasis modules—is consistent with canonical metal-tolerance strategies underpinning root resilience.

For fungi (Fig. 5b), FUNGuild assignments (*P* < 0.05) showed that a broad endophyte–litter/soil saprotroph–undefined saprotroph composite guild constituted a larger share in the mining-impacted rhizosphere, whereas the fungal parasite–litter saprotroph category was relatively higher in the reference rhizosphere. This pattern is consistent with the possibility that contamination selects for saprotrophic and endophytic strategies while disfavoring pathotroph-associated guilds, yielding a microbiome more conducive to crop resilience under metal stress.

### Soil chemistry–microbiome coupling and pathways inferred by SEM

**Fig. 6:**
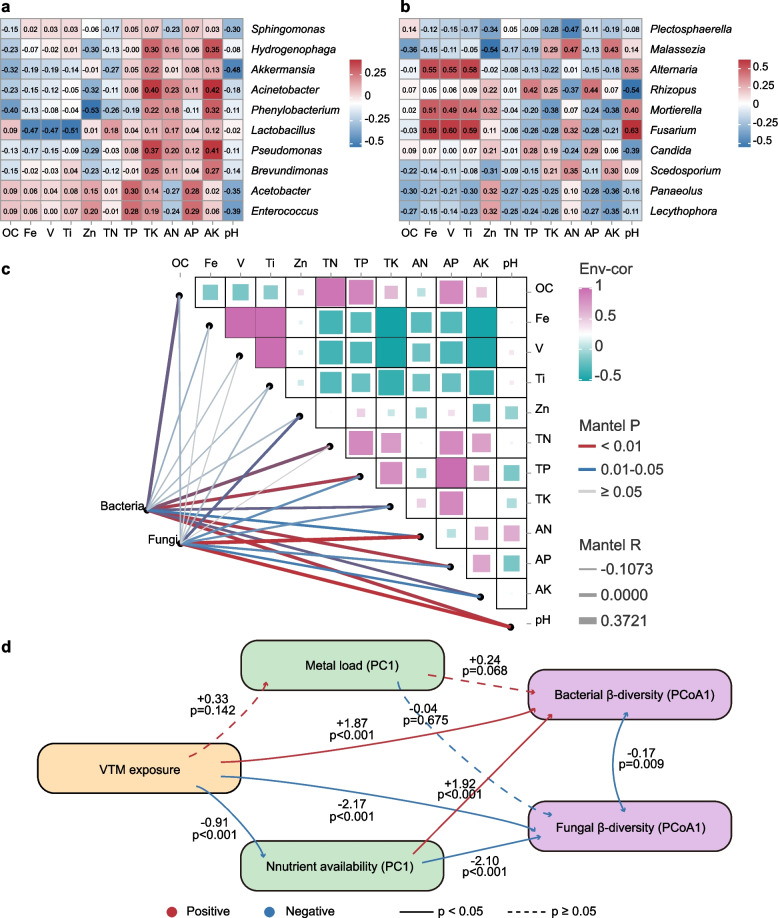
Soil chemistry–microbiome coupling revealed by genus–environment correlations, Mantel tests and structural equation modeling (SEM). **a** Bacteria: Pearson correlation heatmap between the top-10 genera and soil variables (OC, Fe, V, Ti, Zn, TN, TP, TK, AN, AP, AK, pH). Cells display Pearson’s r (− 1 to 1); colors encode direction/magnitude. **b** Fungi: as in (**a**) for the top-10 fungal genera. **c** Integrated Mantel analysis linking community dissimilarities to environmental distances: Bray–Curtis dissimilarity (bacterial and fungal ASV tables) vs. Euclidean distance of z-scored soil variables. Edge color represents Mantel p-value and edge width the Mantel r; the upper-right matrix shows Pearson correlations among environmental variables (“Env-cor”). Genera and species names are italicized in legends. **d** SEM linking VTM exposure, metal load (PC1 of Fe, V, Ti and Zn), nutrient availability (PC1 of AN, AP and AK), and bacterial/fungal community turnover (Bray–Curtis PCoA1 scores). Numbers on paths are standardized coefficients; solid lines indicate significant paths (*P* < 0.05), and dashed lines indicate non-significant paths. OC, soil organic carbon; TN, total nitrogen; TP, total phosphorus; TK, total potassium; AN, alkali-hydrolyzable nitrogen; AP, Olsen-P; AK, available potassium

Based on correlation heatmaps of the top-10 genera and the joint Mantel analysis (Fig. 6), we observed consistent but genus-specific associations between dominant rhizosphere taxa and key soil variables. For bacteria (Fig. 6a), most dominant genera showed weak-to-moderate negative correlations with major metals (Fe, V and Ti), with *Lactobacillus* displaying the strongest negative associations across these elements. In contrast, several genera—including *Acinetobacter*, *Pseudomonas*, *Brevundimonas* and *Hydrogenophaga*—tended to correlate positively with K-related pools (TK and AK) and, to a lesser extent, available P, which could indicate a coupling between nutrient availability and shifts in dominant bacterial groups. For fungi (Fig. 6b), clearer metal- and pH-associated patterns emerged: *Alternaria* and *Fusarium* were strongly positively correlated with Fe/V/Ti (and with pH), while *Mortierella* showed positive associations with multiple metals (including Zn) but negative associations with available P and K. In addition, *Rhizopus* and *Candida* were positively associated with P-related variables (TP and/or AP) yet negatively correlated with pH, whereas *Malassezia* showed positive correlations with pH and nutrient-related variables (notably AN/AK), consistent with niche differentiation along combined chemistry gradients.

Mantel tests (Bray–Curtis community dissimilarities × Euclidean distances of z-scored environmental variables) further demonstrated significant associations between community composition and the environmental matrix (Fig. 6c), with pH showing the strongest overall link to both bacterial and fungal β-diversity (Mantel r in the moderate range, *P* < 0.05); P and K pools (TP, AP, TK) and several metals (V, Ti, Zn, Fe) also exhibited secondary associations. The covariance among environmental variables themselves (e.g., among metals and between OC and TN) suggests that multiple, co-varying gradients jointly drive community differentiation.

Building on these association patterns, we applied SEM to disentangle direct and indirect links among VTM exposure, metal load (PC1 of Fe, V, Ti and Zn), nutrient availability (PC1 of AN, AP and AK), and community compositional shifts represented by Bray–Curtis PCoA1 scores (Fig. 6d). Using PCA-derived composite axes helps summarize correlated soil variables and reduces collinearity in a parsimonious SEM framework [35]. The final model showed a good fit (χ^2^ = 0.33, df = 1, *P* = 0.56; CFI = 1.00; SRMR = 0.024; RMSEA = 0.00). VTM exposure was associated with a higher metal load and markedly reduced nutrient availability, with the nutrient path being stronger. Nutrient availability was a major predictor of microbiome compositional shifts, showing a positive association with bacterial PCoA1 but a negative association with fungal PCoA1, indicating opposite shifts along the primary ordination axis. In addition, VTM exposure also showed significant direct associations with bacterial (positive) and fungal (negative) PCoA1. Metal load exhibited at most a marginal association with bacterial PCoA1 and no clear effect on fungal PCoA1. Notably, bacterial and fungal PCoA1 scores remained negatively correlated after accounting for VTM and environmental predictors, suggesting that community structure may undergo coupled but opposing changes under mining-associated conditions.

## Discussion

### Influence of VTM mining on elements in the surrounding soil

Heavy‐metal pollution of soils is a pervasive global problem with well-documented risks to ecosystem functioning, food safety and human health [36]. Soils from croplands within the VTM mining influence zone showed a clear geochemical imprint relative to the reference fields, characterized by enrichment of Fe, V and Ti (with Zn variably elevated), a neutral–alkaline pH, and pronounced depletion of available P and K in both bulk and rhizosphere compartments (Table S1; Fig. 6c). Among all groups, the pea rhizosphere at the mining site (VT-Psa) exhibited the strongest Fe–V–Ti signal, whereas lettuce and rapeseed showed more moderate or crop-dependent shifts, indicating a steep but plant-modulated edaphic gradient.

Vanadium is a plausible contributor to shaping this gradient. In soils, V commonly occurs as vanadate (V(V)) and vanadyl (V(IV)), and under neutral–alkaline conditions, vanadate can behave analogously to phosphate, competing for sorption sites and biological transport pathways [10, 37]. Such chemistry is consistent with the strong association between pH/vanadium and community composition detected by Mantel tests (Fig. 6c) and with reports from the Panzhihua VTM district documenting substantial V enrichment in agricultural soils affected by tailings, dust, and runoff [2, 5, 38]. In contrast, titanium generally has low mobility and phytoavailability because it is bound in resistant minerals and poorly soluble oxides [39, 40]; therefore, we interpret Ti here primarily as a tracer of mining-derived particulate inputs that co-deposit with Fe and V, consistent with the coherent Ti–V–Fe block in our correlation matrix (Fig. 6c).

Nutrient depletion likely represents an additional pathway through which mining disturbance may constrain the rhizosphere. The marked reductions in available P and K in mining-site rhizospheres (Table S1) likely reflect enhanced P retention/precipitation in alkaline, Fe-rich matrices and reduced effective nutrient cycling under metal stress, together establishing a combined metal–pH–nutrient gradient that can shape rhizosphere microbiome assembly; notably, nutrient limitation emerged as an important route in our SEM framework (Fig. 6d).

Although V–Ti magnetite mining environments can involve co-occurring trace elements in regional particulates (e.g., Cr, Mn, Ni, Pb) [41, 42], our geochemical profiling focused on Fe–V–Ti–Zn; therefore, the “metal load” discussed here should be interpreted as a measured Fe–V–Ti–Zn composite rather than a comprehensive multi-metal pollution index. Vanadium stress alone has been shown to alter soil microbial activities and community structure in controlled incubations, supporting the plausibility that V-associated stress contributes to microbiome reorganization [43]. Given that co-occurring metals can impose additional or interactive constraints on soil microbiota (e.g., Cr–V co-release scenarios), future work should extend multi-element measurements and explicitly test mixture effects. In addition, although we selected paired fields with comparable routine practices, unmeasured fine-scale differences in management intensity (e.g., fertilization history, irrigation frequency, or pesticide inputs) cannot be fully excluded, which is a common limitation of field-based microbiome studies.

### Effects of VTM mining on rhizosphere microbial communities

Across crops, VTM exposure reshaped rhizosphere microbiomes in a host-dependent yet environmentally constrained manner. Alpha diversity shifted idiosyncratically—declining in lettuce but increasing markedly in pea and (for bacteria) rapeseed (Fig. 1; Fig. S2)—whereas beta diversity (Bray–Curtis PCoA/NMDS) consistently separated mining from reference rhizospheres and further resolved crop-specific subclusters (Fig. 2; Fig. S3). Together, these patterns are consistent with a contamination filter whose effects are modulated by plant identity. Such crop-specific trajectories may reflect differences in rhizosphere niche construction. Legumes can recruit distinctive root-associated consortia via nodulation and N-related niche processes, which can reorganize microbial assembly in model legumes such as *Lotus japonicus* [44]. Moreover, symbiosis signaling (e.g., Nod-factor pathways) can shape root-associated microbiota assembly beyond nitrogen inputs, providing a mechanistic basis for host-contingent trajectories under shared edaphic constraints [45]. Brassicaceae rhizospheres are also strongly influenced by sulfur-rich specialized metabolites; genetic and pathway-level evidence indicates that specialized-metabolite networks can selectively modulate root microbiota composition [46–48]. For leafy vegetables, metal transfer to edible tissues is a recurring food-safety concern, and lettuce is often used as an indicator crop in metal-exposure studies [48].

At the taxonomic level, rhizosphere bacterial communities were consistently dominated by Proteobacteria, while fungi were overwhelmingly dominated by Ascomycota across all treatments (Fig. 3a, c). At the genus level, *Sphingomonas* remained relatively abundant under different conditions, whereas several bacterial genera enriched in reference fields (e.g., *Hydrogenophaga*, *Pseudomonas*) declined under mining influence (Fig. 3b). Fungal communities exhibited pronounced, host-dependent turnover (Fig. 3d). Lettuce shifted toward *Panaeolus* and *Lecythophora*; rapeseed showed increases in *Plectosphaerella* and *Mortierella*, while pea displayed a complex shift involving increases in multiple genera (e.g., *Plectosphaerella*, *Alternaria*, *Fusarium*, *Mortierella*) and decreases in others (e.g., *Malassezia*, *Scedosporium*). LEfSe analysis further supported these shifts: mining-exposed rhizospheres were characterized by stress-resistant bacterial clades such as Actinobacteriota and fungal indicators including *Mortierella* and *Panaeolus*, whereas reference fields were distinguished by Pseudomonadales and *Malassezia* (Fig. 4), indicating systematic community restructuring under metal stress.

To further connect the LEfSe-identified biomarkers with community-level organization, we inferred genus-level co-occurrence networks for dominant bacterial and fungal genera (Fig. 4e–h). Compared with reference rhizospheres, the mining-impacted networks displayed a re-arranged modular structure and a higher predominance of positive associations, while overall connectivity tended to decrease (Table S4), consistent with stronger environmental filtering and more compartmentalized co-variation patterns among dominant taxa under combined metal enrichment and nutrient limitation [49]. Consistent with ordination patterns, a two-factor PERMANOVA confirmed a significant plant × contamination interaction (Tables S2–S3), supporting crop-dependent compositional responses to VTM exposure under shared environmental constraints. Notably, correlation-based co-occurrence reflects statistical co-variation and does not by itself demonstrate direct biotic interactions [50]. Nevertheless, these network- and interaction-level inferences provide useful hypotheses and practical targets for follow-up validation in future work, for example, via targeted qPCR/dPCR assays and isolate-based characterization.

### Functional reprogramming and plant–microbe–soil feedbacks under metal stress

Integrating predictive functions (PICRUSt2, bacteria) and guild assignments (FUNGuild, fungi) with the measured environmental gradients (Figs. 5 and 6) is consistent with a potential coordinated shift from resource-acquisition toward stress-accommodation strategies in rhizosphere microbiomes exposed to VTM-site metals. On the bacterial side (Fig. 5a), reference fields were relatively enriched for carbohydrate-use modules (e.g., glycoprotein metabolism, PTS-mediated sugar uptake), whereas VTM rhizospheres showed higher representation of stress and envelope processes (e.g., responses to divalent-ion limitation, D-amino-acid transport, positive regulation of lipid biosynthesis)—a trade-off consistent with canonical metal-tolerance toolkits (membrane remodeling, efflux/chelation systems, and oxidative-stress mitigation) [51]. Mantel tests and genus–environment correlations (Fig. 6) identify pH as the dominant selector—with P/K pools and Fe/V/Ti/Zn as secondary axes—which helps explain host-specific trajectories of copiotrophs: for example, *Sphingomonas* tended to maintain or increase where pH and AP were higher, aligning with its reported rhizosphere competence and abiotic-stress/metal-tolerance traits that support plant performance under challenging edaphic conditions [52]. Fungal guilds were also rebalanced under contamination (Fig. 5b): mining-impacted rhizospheres favored generalist endophyte–/litter-/soil-saprotroph composites, whereas control fields carried relatively more parasite-associated saprotrophs. This pattern aligns with the strong Ascomycota dominance observed here and with reports that saprotrophic/endophytic strategies better persist under nutrient–ion imbalances and metal-induced oxidative pressure.

Together with the crop-specific α/β-diversity outcomes (Figs. 1 and 2; Figs. S2–S3), these patterns are compatible with a plant-mediated filtering model: host exudation and ion- homeostasis needs under metal stress select microbial partners equipped with stress-response machinery, while pH and macronutrient availability constrain the feasible taxonomic/functional solution space [20, 53]. Collectively, our data suggest that metal exposure at the VTM site may rewire rhizosphere microbiomes toward stress-tolerant, membrane/transport/ROS-response-enriched configurations, with plant identity and soil chemistry jointly determining which bacterial and fungal lineages and which functions are favored. As a next step, targeted enzyme assays (phosphatase/urease/dehydrogenase), shotgun metagenomics/metatranscriptomics combined with targeted isolate assays would help validate these inferred functions.

Building on the correlation and Mantel results, SEM provided a parsimonious framework linking VTM exposure, metal load, nutrient availability, and community compositional shifts (ordination scores) (Fig. 6d). VTM exposure was strongly associated with reduced nutrient availability, whereas its association with the composite metal-load axis was weaker. Nutrient availability emerged as a major predictor of compositional shifts, showing opposite associations with bacterial vs fungal ordination scores, suggesting divergent kingdom-level responses along the primary beta-diversity axis. Metal load showed at most a marginal relationship with bacterial ordination scores and no clear effect on fungal scores. Notably, we quantified total metals rather than bioavailable fractions; bioavailability can diverge from total concentrations and is strongly controlled by soil properties (e.g., pH, organic matter, and sorption/precipitation), especially in neutral–alkaline systems [54, 55]. Thus, the weak SEM path from total-metal load to community turnover does not preclude the effects of bioavailable metals, which should be evaluated using DTPA-extractable or pore-water metals in future work. Finally, bacterial and fungal ordination scores retained a negative residual association after accounting for environmental predictors, indicating partially coupled but directionally different reorganization under mining-associated conditions; however, this residual covariance should be interpreted as shared unexplained variation (e.g., unmeasured bioavailable metal fractions, redox/ionic conditions, or host-specific rhizosphere chemistry) rather than evidence for direct biotic antagonism.

### Implications for microbially assisted remediation and management

From a crop-health perspective, our results highlight two potentially complementary levers: assembling host-compatible, stress-tolerant consortia and applying abiotic steering to stabilize pH and rebuild plant-available P/K. This prioritization is supported by the strong environment–microbiome coupling and the SEM framework, in which nutrient availability emerged as a major pathway linking VTM exposure to community turnover (Fig. 6). Evidence from phosphate-mining districts—where Cd/Pb co-enrichment coincides with higher relative abundances of *Sphingomonas* and RB41—highlights realistic bioremediation candidates [19, 56]. Concordantly, in the VTM context, *Sphingomonas* persisted across lettuce and pea, whereas several genera prevalent in the reference field (e.g., *Hydrogenophaga*, *Acinetobacter*, *Pseudomonas*, and *Brevundimonas*) declined in mining-site rapeseed and pea rhizospheres (Figs. 3 and 4), consistent with stress- and nutrient-driven filtering. On the fungal side, *Mortierella* is a plausible ally given reported plant-growth-promoting and nutrient-mobilizing traits [29], whereas tolerant/opportunistic groups (e.g., *Lecythophora*, *Scedosporium*) suggest that remediation gains should be balanced against potential plant-health risks [57].

Joint bacterial (PICRUSt2-inferred functions) and fungal (FUNGuild assignments) profiling revealed complementary shifts—bacterial communities relatively enriched in stress/envelope processes and fungi tilted toward saprotrophic/endophytic guilds (Fig. 5)—which can help nominate candidate inoculants and define a concise monitoring panel for VTM-impacted soils [29, 58]. In addition, the co-occurrence networks suggest a reorganization of association patterns among dominant taxa under mining-associated gradients, consistent with broad community reassembly rather than isolated taxon changes (Fig. 4e–h).

Translating these insights into management, pH-conscious amendments and P/K restoration could be prioritized to increase the likelihood that selected partners establish and function, consistent with our Mantel results identifying pH (with P/K) as the dominant template shaping communities (Fig. 6). Overall, crop–microbe–soil interactions in VTM-impacted fields may be leveraged by pairing suitable hosts with differentiated, high-tolerance strains tailored to the rhizosphere context, while policy instruments remain essential to curb mining-related damage, support long-term monitoring, and sustain phased restoration [11, 56].

## Conclusions

VTM mining left a distinct geochemical signature in adjacent cropland soils, including Fe–V–Ti enrichment in bulk soils, neutral–alkaline pH, and depleted plant-available P and K. Across crops, Bray–Curtis β-diversity consistently separated mining-impacted from reference rhizospheres, whereas α-diversity responses were host contingent. Bacterial communities remained Proteobacteria-dominated, with *Sphingomonas* remaining prominent across hosts; several genera prevalent in reference rhizospheres (e.g., *Pseudomonas*, *Acinetobacter*, *Hydrogenophaga*) tended to decline under mining impact, while lettuce showed increases in *Akkermansia* and *Phenylobacterium*. Fungal assemblages were Ascomycota-dominated but exhibited host-specific turnover, including higher *Panaeolus*/*Lecythophora* in lettuce and higher *Mortierella* in rapeseed and pea (with pea also showing increased *Alternaria*/*Fusarium*). Mantel tests linked community turnover mainly to covarying pH and nutrient status, and SEM was consistent with reduced nutrient availability (AN/AP/AK composite) as a major pathway, with only weak additional contributions from the composite total metal-load axis (Fe/V/Ti/Zn). Relative to previous studies, we emphasize the rhizosphere niche, paired two-kingdom profiling, and SEM-based path decomposition to help disentangle nutrient-mediated versus metal-load effects, thus providing a basis to assess crop-health risk and to deploy plant-aware, microbiome-assisted remediation in metal-impacted croplands.

## Materials and methods

### Study area and sample collection

This study was conducted around the Hongge South VTM district near Hongge Town, Yanbian County, Panzhihua, Sichuan, China (~ 26°32′–26°42′ N, 101°56′–101°58′ E). The region has a monsoon-influenced humid subtropical climate (Köppen Cwa), with a mean annual temperature of approximately 19–20 °C and ~ 850–1100 mm of precipitation, most of which falls from May/June to October. Mining and smelting have been active for decades, generating mine pits, waste rock and slag deposits; windblown dust and surface runoff can redistribute metal-rich particulates into adjacent lands. The geographic coordinates and site metadata for all sampling locations are provided in Fig. S1.

Croplands in the Panzhihua dry–hot valley are predominantly developed on purple-soil-derived parent materials and typically show loam to clay-loam textures with relatively high silt and clay fractions. Because soil texture and field management can influence metal partitioning and microbial assembly, we adopted a paired-field design in which the same crop types were sampled in a mining-influenced area and in a reference area, and we focused on rhizosphere soils to reduce background heterogeneity. The sampled fields were managed under conventional local agricultural practices; within each crop, paired fields were selected to be comparable in routine management (e.g., fertilization regime and irrigation practice) as far as practical to minimize non-mining confounding.

We sampled two zones: (i) a mining-influenced area adjacent to the Hongge South VTM district and (ii) a reference cropland outside the mining influence. From each zone, we collected rhizosphere soils of three locally grown crops—lettuce (*Lactuca sativa* cv. Italy Lettuce; Lsa/VT-Lsa), rapeseed (*Brassica rapa* cv. Yunyouza 15; Bra/VT-Bra), and pea (*Pisum sativum* cv. Zhongwan 6; Psa/VT-Psa)—as well as bulk soils without crop roots (CK at the reference site; VT-CK at the mining site) to provide a geochemical context. For every group, three biological replicates were obtained (independent plants/plots per crop and site). These crops were chosen to represent distinct functional host types (leafy vegetables, crucifers, legumes) that are expected to differ in rhizosphere processes such as secondary metabolite exudation and N-related niches.

Rhizosphere soil was collected following a standard operational definition. Briefly, whole plants were carefully uprooted; loosely adhering soil was gently shaken off, and the soil still tightly attached to fine roots (approximately within 1–2 mm of the root surface) was removed using a sterile brush and designated rhizosphere soil. Bulk soils (CK and VT-CK) were taken from nearby topsoil at locations without plant roots within the same fields. All samples were placed in sterile containers, transported to the laboratory on ice, and subsequently processed for DNA extraction and high-throughput amplicon sequencing (16S rRNA for bacteria; ITS for fungi).

### Soil physicochemical analysis

For each soil sample, routine physicochemical properties and total metal contents were determined on air-dried, homogenized subsamples and reported on a dry-weight basis. Soil pH was measured with a glass electrode in a soil–water suspension (1:2.5, w/v) (NY/T 1121.2–2006). Soil organic carbon (SOC) was quantified by dichromate oxidation with the external heating (Walkley–Black) method (NY/T 1121.6–2006). Total nitrogen (TN) was measured after H₂SO₄ digestion with a catalyst (Kjeldahl method) (LY/T 1228–2015). Total phosphorus (TP) and total potassium (TK) were determined by NaOH alkaline fusion; TP was read by molybdenum–antimony colorimetry (UV–Vis) (GB/T 9837–1988), and TK was read by flame photometry (NY/T 87–1988). Alkali-hydrolysable (available) nitrogen (AN) was measured using the alkaline diffusion method (LY/T 1229–1999). Available phosphorus (AP) was extracted with 0.5 M NaHCO₃ (Olsen’s method) and determined by molybdenum–antimony colorimetry (NY/T 1121.7–2014). Available potassium (AK) was extracted with 1.0 M ammonium acetate and determined by flame photometry (NY/T 889–2004).

We focused on Fe, V and Ti as signature elements of V–Ti magnetite mining/processing inputs and included Zn as a common co-occurring trace metal; other potentially relevant metals (e.g., Cr, Mn, Pb) were not quantified in the present dataset. For total metals, ~ 0.5 g soil was subjected to four-acid digestion, and the digest was analyzed by inductively coupled plasma‒mass spectrometry (ICP-MS) to quantify Fe, V, Ti and Zn. Calibration employed multi-element standards; routine QA/QC included reagent blanks, replicate digestions, and certified reference materials (DZT 0279.2–2016). All measurements were performed on three biological replicates per group (independent field replicates).

### DNA extraction and 16S-V3/V4 & ITS2 amplicon sequencing

Microbial DNA was extracted from ~ 0.5 g of fresh rhizosphere/bulk soil per sample using the HiPure Soil DNA Kit B (Magen, China) following the manufacturer’s instructions. DNA quantity was measured with a Qubit 3.0 fluorometer using the dsDNA HS Assay Kit; purity and integrity were checked by agarose gel electrophoresis. For each sample, 20–30 ng of DNA served as a template for two amplicon targets: the bacterial 16S rRNA gene V3–V4 region and the fungal ITS2 region.

Amplicon PCRs were performed in technical triplicate per sample with provider-validated primer sets for 16S-V3/V4 and ITS2, alongside no-template negative controls. PCR conditions followed the kit/provider recommendations for high-fidelity amplification. Triplicate reactions were pooled per sample; amplicons were purified using standard magnetic-bead cleanup and quantified by Qubit. Dual-indexed libraries were prepared according to the Illumina workflow, verified for expected size by gel electrophoresis, normalized to equimolar concentrations, and pooled. Sequencing was carried out on an Illumina MiSeq platform.

### Amplicon sequence processing and taxonomic assignment

Paired-end raw reads were merged by overlap, quality-filtered, and screened for chimeras to obtain high-quality clean reads. Denoising was performed with DADA2 to infer amplicon sequence variants (ASVs) at single-nucleotide resolution, generating a feature (ASV) table and representative sequences for downstream analyses.

For 16S V3–V4 data, taxonomic assignment was conducted against the SILVA reference database (release 138) and an NT-16S reference set using a naïve Bayes classifier (confidence ≥ 0.70). ASVs annotated as chloroplasts or mitochondria were removed prior to ecological analyses. For ITS2 data, taxonomic assignment used the RDP classifier trained on the UNITE database (confidence ≥ 0.70), and non-fungal ASVs were discarded. From the annotated ASV table, taxon abundance tables were generated at the domain through species levels for community composition analyses.

### Alpha- and beta-diversity analyses

Alpha diversity was computed on the rarefied ASV table, including observed ASV richness, Chao1, Shannon, Simpson, and Pielou’s evenness. Between-group differences (e.g., mining vs. reference; crop identity) were evaluated after checking normality (Shapiro–Wilk) and variance homogeneity (Levene). When assumptions were met, one-way ANOVA (or two-way ANOVA where applicable) with Tukey’s HSD post hoc test was used; otherwise, the Kruskal–Wallis test with Dunn’s post hoc test was applied. P values for multiple pairwise tests were adjusted by the Benjamini–Hochberg FDR procedure.

Beta diversity was assessed from Bray–Curtis dissimilarities computed on relative-abundance data (square-root transformed to down-weight dominant taxa). Ordinations used PCoA and NMDS (metaMDS, k = 2, multiple random starts). Group separation was tested with PERMANOVA (adonis2, 9,999 permutations) using a model including contamination status, crop, and their interaction (community ~ contamination * crop); we report R^2^ and permutation *P* values. The homogeneity of multivariate dispersion among groups was examined with betadisper/permutest to guard against dispersion-driven artifacts. When useful for visualization, hierarchical clustering (UPGMA) of Bray–Curtis distances was also generated.

### Differential abundance (LEfSe) for mining vs. reference rhizospheres

To identify biomarkers consistently associated with mining impact across host plants, we performed a pooled rhizosphere biomarker analysis. “Pooling” here means that all planted rhizosphere samples were combined into one analysis set without averaging while retaining each biological replicate as an independent sample (three crops × two field types × three replicates). Bulk soils (CK, VT-CK) were analyzed separately and not mixed with rhizospheres to avoid niche confounding and because of their higher dispersion [59]. Meanwhile, because pooled biomarker discovery emphasizes signals that are consistent across host plants, crop-specific responses may be under-detected (potential false negatives) [31, 60]; therefore, pooled biomarkers were interpreted as cross-host indicators and complemented by crop-stratified community descriptions (Fig. 3) and interaction tests (PERMANOVA; Tables S2–S3).

Starting from the rarefied ASV table, counts were converted to relative abundance and aggregated to multiple taxonomic ranks (phylum → class → order → family → genus; species where confidently assigned). Extremely rare features (total abundance < 0.01% across all rhizosphere samples or present in < 10% of samples) were filtered to reduce spurious calls. LEfSe was run with contamination status as the class and crop (lettuce/rapeseed/pea) as the subclass to account for host stratification during the Wilcoxon step; default nonparametric tests (Kruskal–Wallis across classes, followed by pairwise Wilcoxon across subclasses) and linear discriminant analysis were used. Significance thresholds were set to α = 0.05 (two-sided) and LDA (log10) ≥ 3.0. The results are reported as clade-consistent biomarkers with direction (enriched in mining-impacted rhizosphere or reference rhizosphere) and effect size (LDA score).

### Co-occurrence network analysis

To explore potential associations among dominant microbial taxa, we constructed co-occurrence networks for both bacterial and fungal communities. ASVs were first aggregated to the genus level, and the top 10 most abundant genera were retained for each kingdom as the basis for network analysis. ASV filtering and Spearman correlation calculations were performed in R (version 4.5.2). Pairwise Spearman correlation coefficients were calculated between genera across samples, and only associations with |r|≥ 0.70 and *P* < 0.05 were considered significant and included in the networks. Co-occurrence networks were visualized in Gephi (version 0.10.1), with edge color denoting positive or negative correlations and node size scaled to mean relative abundance of the corresponding genus.

### Functional prediction and bioinformatic analysis

Functional inference. For bacterial 16S profiles, predicted functional potentials were generated with PICRUSt2 [61] and mapped to the Gene Ontology—Biological Process (GO-BP) terms. For fungal ITS profiles, ecological functions were assigned using FUNGuild (database v1.0; [62]). Fungal ASVs with “highly probable” or “probable” confidence were retained and categorized into trophic modes (pathotroph, symbiotroph, saprotroph) and associated guilds; unassigned entries were labeled “unknown”. Relative abundances of each trophic mode per sample were computed by summing the reads (or relative frequencies) of member ASVs.

Between-group comparisons (STAMP). Predicted bacterial GO-BP terms and fungal trophic/guild compositions were compared in STAMP [63] between reference rhizospheres and mining-impacted rhizospheres. For these pooled comparisons, all planted rhizosphere samples from the three crops were grouped by contamination status, while bulk soils (CK and VT-CK) were excluded to avoid niche confounding. Biological replicates were retained as independent samples (i.e., not averaged). Unless otherwise noted, nonparametric tests (Wilcoxon rank-sum test) were applied with *p* ≤ 0.05, and the results are reported with effect sizes and 95% confidence intervals. Because pooling across crops is intended to capture overall status-associated tendencies, it may reduce sensitivity to crop-specific signals (potential false negatives) when responses differ among hosts; therefore, pooled STAMP results are interpreted as shared trends and are complemented by crop-resolved community analyses reported elsewhere. Where count-based differential abundance was needed, metagenomeSeq was used with thresholds *p* = 0.05 and |log2FC|= 0.

Dissimilarities and Mantel tests. Community compositional dissimilarities were calculated as Bray–Curtis distances using vegdist in the vegan R package. To relate microbiome metrics to the environment, Mantel tests were conducted using Mantel (vegan). Unless specified, bioinformatic analyses and visualizations were carried out in OmicStudio [64] and R (version 4.5.2).

### SEM

SEM was used to quantify the direct and indirect effects of VTM exposure on the microbial community structure. Metal load (Fe, V, Ti, Zn) and nutrient availability (AN, AP, AK) were summarized as PC1 scores from PCA on z-standardized variables. Bacterial and fungal community structure was represented by PCoA1 scores derived from Bray–Curtis dissimilarity matrices of 16S and ITS ASV tables. The SEM included paths from VTM to the two environmental indices and to microbial PCoA1, with additional paths from environmental indices to microbial PCoA1 and a covariance between bacterial and fungal PCoA1. Given the limited rhizosphere sample size available for SEM (*n* = 18), we specified a parsimonious model and did not include crop identity as an additional grouping or moderating factor to avoid over-parameterization and unstable estimation [65]. Models were fitted by maximum likelihood and evaluated using χ^2^, CFI/TLI and RMSEA [66].

## Supplementary Information

Supplementary Material 1: Fig. S1. Study area and sampling design around the Hongge VTM district (Yanbian County, Panzhihua, Sichuan, China). Fig. S2. Additional alpha-diversity indices and rarefaction curves. Fig. S3. Additional beta diversity of rhizosphere microbiomes. Table S1. Physicochemical properties of soils from the vanadium–titanium magnetite (VTM) mining area and the reference site. Table S2. Two-factor PERMANOVA (Bray–Curtis) of bacterial rhizosphere community composition: effects of crop type, VTM exposure, and their interaction. Table S3. Two-factor PERMANOVA (Bray–Curtis) of fungal rhizosphere community composition: effects of crop type, VTM exposure, and their interaction. Table S4. Network topological indices for bacterial and fungal co-occurrence networks under reference vs VTM-impacted conditions. Data S1. Summary statistics of bacterial and fungal sequencing data. Data S2. Summary statistics of bacterial ASV feature sequences by sample. Data S3. Summary statistics of fungal ASV feature sequences by sample. Data S4. LEfSe-identified differentially abundant taxa (bacteria and fungi) in crop rhizospheres (mining-impacted rhizospheres vs. reference rhizospheres).

## Data Availability

Data will be made available on request.
