# Supplementary material for: Toward microbiome-assisted remediation: Vanadium–titanium magnetite mining reshapes cropland soil chemistry and rhizosphere microbiomes
Source: Crop Health. 2026 Apr 2;4(1):10. doi: 10.1007/s44297-026-00072-9 (PMC13043986; doi:10.1007/s44297-026-00072-9)
Supplement: Supplementary file 1 — Supplementary Material 1: Fig. S1. Study area and sampling design around the Hongge VTM district (Yanbian County, Panzhihua, Sichuan, China). Fig. S2. Additional alpha-diversity indices and rarefaction curves. Fig. S3. Additional beta diversity of rhizosphere microbiomes. Table S1. Physicochemical properties of soils from the vanadium–titanium magnetite (VTM) mining area and the reference site. Table S2. Two-factor PERMANOVA (Bray–Curtis) of bacterial rhizosphere community composition: effects of crop type, VTM exposure, and their interaction. Table S3. Two-factor PERMANOVA (Bray–Curtis) of fungal rhizosphere community composition: effects of crop type, VTM exposure, and their interaction. Table S4. Network topological indices for bacterial and fungal co-occurrence networks under reference vs VTM-impacted conditions. Data S1. Summary statistics of bacterial and fungal sequencing data. Data S2. Summary statistics of bacterial ASV feature sequences by sample. Data S3. Summary statistics of fungal ASV feature sequences by sample. Data S4. LEfSe-identified differentially abundant taxa (bacteria and fungi) in crop rhizospheres (mining-impacted rhizospheres vs. reference rhizospheres). [file 44297_2026_72_MOESM1_ESM.zip › Supplemental materials-20260217.pdf]

Supplemental materials

Toward microbiome-assisted remediation: Vanadium–titanium magnetite mining reshapes cropland soil chemistry and rhizosphere microbiomes

Bingliang Liu[1, †, *], Xiao Huang[1, †], Cheng Chang[1], Xin Wan[1], Mingrong Liu[1], Rui Li[1, 2], Jun Li[3], Qiang Li[1], and Yang Tao[1, 2, *]

[1] College of Food and Biological Engineering, Chengdu University, Chengdu, China;

[2] Sichuan-Xizang Medicinal Resource Breeding and Standardization Team, Institute for Advanced Study, Chengdu University, Chengdu, China;

[3] School of Vanadium and Titanium, Panzhihua University, Panzhihua, China

[†] Bingliang Liu and Xiao Huang contributed equally to this work.

[*] Correspondence to: Yang Tao: taoyang@cdu.edu.cn; Bingliang Liu: liubingliang@cdu.edu.cn

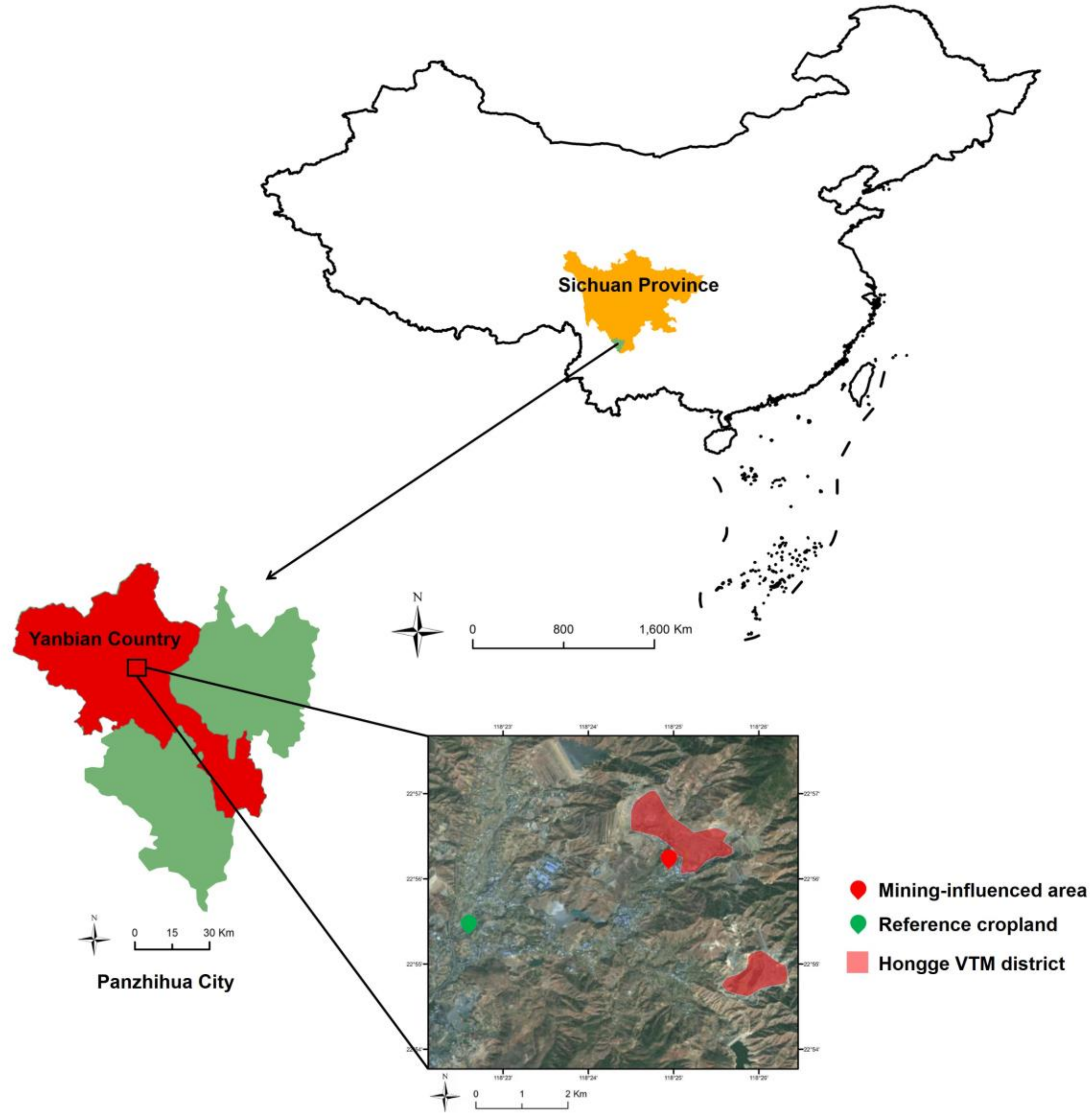

Fig. S1. Study area and sampling design around the Hongge VTM district (Yanbian County, Panzhihua, Sichuan, China).

Two field types were surveyed: (i) mining-influenced fields adjacent to the Hongge VTM district and (ii) reference croplands outside the district. From each field type, we collected rhizosphere soils of three locally cultivated crops—lettuce (*Lactuca sativa*; Lsa / VT-Lsa), rapeseed (*Brassica rapa*; Bra / VT-Bra), and pea (*Pisum sativum*; Psa / VT-Psa)—and bulk soils without plant roots (reference bulk, CK; mining-influenced bulk, VT-CK). Red polygons indicate the Hongge VTM district; red points mark mining-influenced sampling sites; green points mark reference croplands.

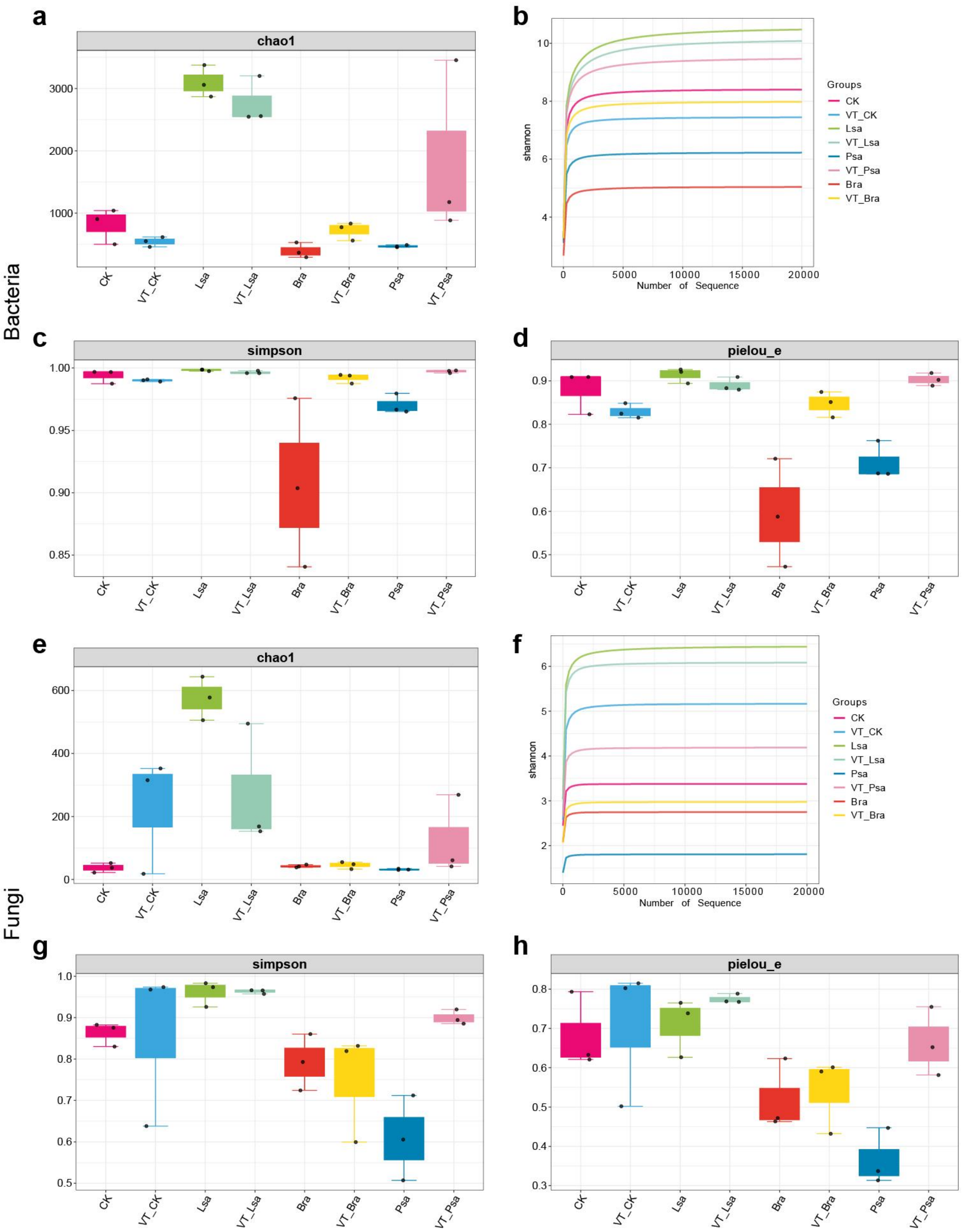

Fig. S2. Additional alpha-diversity indices and rarefaction curves.

Supplementary panels show Chao1 richness, Simpson diversity, and Pielou's evenness for bacterial and fungal communities, together with Shannon rarefaction curves for each group. Lsa, *Lactuca sativa*; Bra, *Brassica rapa*; Psa, *Pisum sativum.*

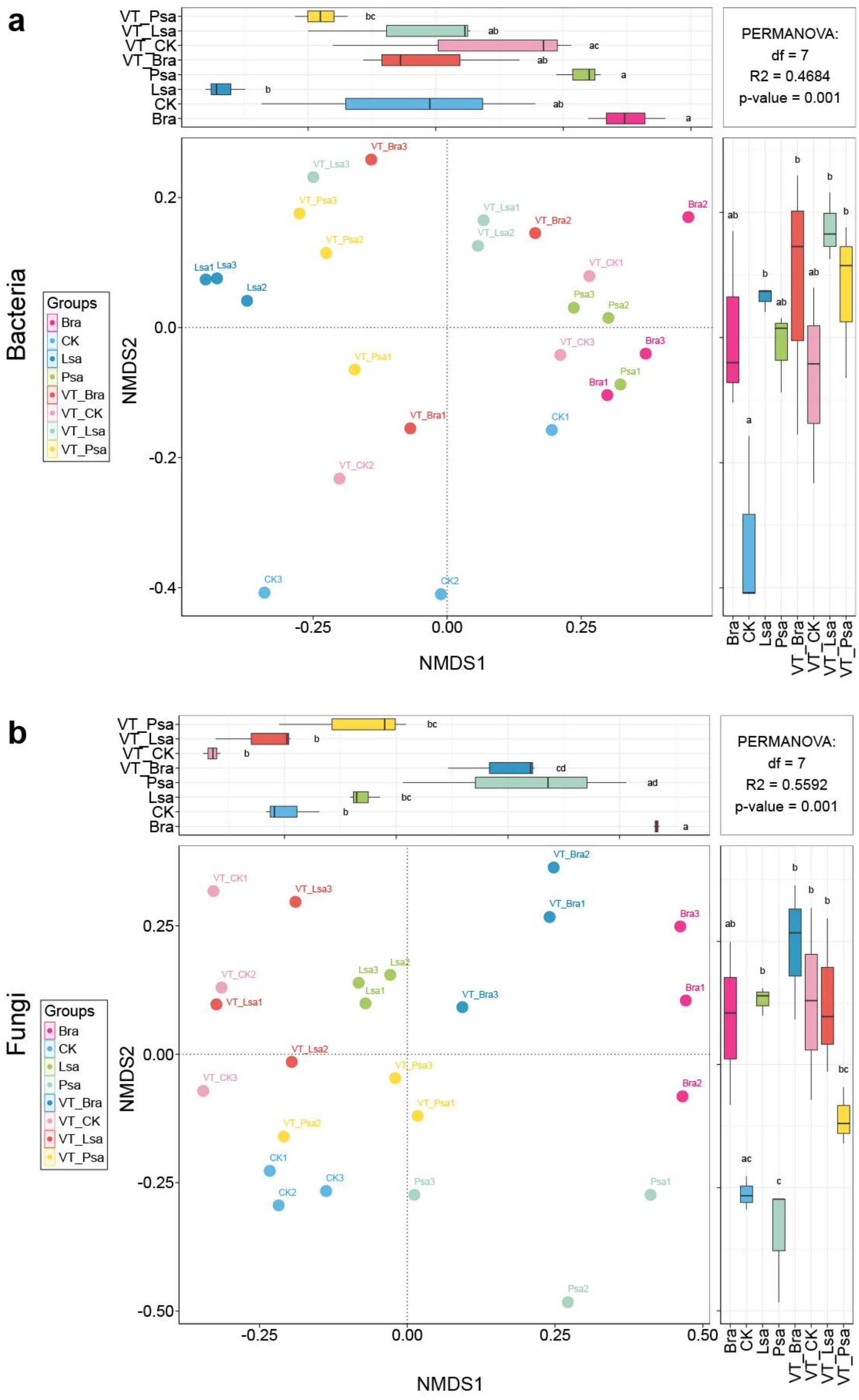

Fig. S3. Additional beta diversity of rhizosphere microbiomes.

(**a**) Bacteria; (**b**) Fungi. Ordinations are based on Bray–Curtis dissimilarities using non-metric multidimensional scaling (NMDS) (stress shown on plot). Points are biological replicates;

colors/shapes denote treatment groups (CK, VT-CK, Lsa, VT-Lsa, Bra, VT-Bra, Psa, VT-Psa). The top and right-side boxplots summarize group distributions of the corresponding axis scores; gray insets report PERMANOVA results (vegan adonis2: R^2 and p-value). Lsa, *Lactuca sativa*; Bra, *Brassica rapa*; Psa, *Pisum sativum.*

Table S1. Physicochemical properties of soils from the vanadium–titanium magnetite (VTM) mining area and the reference site

Element	VT-CK	CK	VT-Lsa	Lsa	VT-Bra	Bra	VT-Psa	Psa
pH	7.97±0.04a	7.05±0.26g	7.36±0.01e	7.80±0.03c	7.30±0.02f	7.01±0.01g	7.88±0.04b	7.65±0.06d
OC (g/kg)	2.85±0.10h	3.40±0.11g	4.22±0.10e	11.74±0.27a	5.67±0.15d	7.18±0.22b	6.03±0.28c	3.88±0.20f
Fe (g/kg)	45.26±1.01d	34.03±0.75f	44.90±1.57d	41.08±0.26e	48.63±0.70c	57.93±1.76b	90.96±2.89a	46.56±0.63dc
V (mg/kg)	105.00±2.64e	87.33±3.78f	114.66±2.51d	102.33±3.51e	124.00±3.46c	160.66±9.07b	302.33±5.85a	129.33±6.80c
Ti (g/kg)	4.60±0.20ef	4.20±0.10f	4.50±0.10f	5.06±0.57e	5.86±0.28d	9.26±0.25b	17.36±0.60a	7.10±0.10c
Zn (mg/kg)	67.66±2.08c	69.00±0.26c	107.66±2.88b	101.66±5.50b	70.00±2.00c	102.66±2.51b	97.00±3.60b	67.00±3.60c
TN (g/kg)	0.50±0.10g	0.54±0.00f	0.55±0.00e	1.27±0.01a	0.61±0.00d	0.74±0.01b	0.60±0.00d	0.63±0.00c
TP (g/kg)	0.76±0.01e	1.26±0.03c	0.83±0.02d	1.95±0.02a	0.83±0.02d	1.77±0.02b	0.82±0.01d	0.85±0.01d
TK (g/kg)	14.56±0.25d	23.96±0.15a	13.10±0.20f	20.50±0.26b	12.06±0.15g	18.26±0.15c	11.46±0.11h	18.10±0.26c
AN (mg/kg)	48.66±1.15h	102.66±2.08e	125.33±0.57c	143.00±1.00b	68.66±0.57g	81.33±2.08f	110.00±3.00d	160.33±1.52a
AP (mg/kg)	4.13±0.20f	177.86±4.54a	13.63±0.68e	168.53±6.77b	18.10±0.26e	149.23±6.92c	12.73±0.41e	32.66±1.26d
AK (mg/kg)	67.66±1.52g	219.00±0.00d	75.66±1.52f	427.33±4.16a	116.66±3.05e	256.66±4.04c	70.00±0.00fg	390.66±6.80b

OC, soil organic carbon; TN, total nitrogen; TP, total phosphorus; TK, total potassium; AN, alkali-hydrolyzable nitrogen; AP, Olsen-P; AK, available potassium. Sample codes: VT-CK, bulk soil at the VTM site (uncultivated); CK, bulk soil at the reference site (uncultivated); Lsa, *Lactuca sativa*; Bra, *Brassica rapa*; Psa, *Pisum sativum.* Values are means ± SE (n = 3). Different lowercase letters within a row indicate significant differences among treatments (LSD, $P < 0.05$).

Table S2. Two-factor PERMANOVA (Bray–Curtis) of bacterial rhizosphere community composition: effects of crop type, VTM exposure, and their interaction

Term	F	df	R^2	*p*
Crop	2.088949	2	0.177966	0.001
VTM	2.67046	1	0.113754	0.002
Crop × VTM	2.3137	2	0.197114	0.001

Table S3. Two-factor PERMANOVA (Bray–Curtis) of fungal rhizosphere community composition: effects of crop type, VTM exposure, and their interaction

Term	F	df	R^2	*p*
Crop	2.37468	2	0.240482	0.001
VTM	2.274686	1	0.106157	0.001
Crop × VTM	2.443068	2	0.189055	0.001

Table S4. Network topological indices for bacterial and fungal co-occurrence networks under reference vs VTM-impacted conditions

Core topological indices	Bacteria		Fungi	
	Reference rhizospheres	Mining-impacted rhizospheres	Reference rhizospheres	Mining-impacted rhizospheres
Nodes	214	323	86	83
Edges	4676	4119	740	394
Density	0.205	0.079	0.202	0.116
Average degree	43.701	25.505	17.209	9.494
Average clustering coefficient	0.805	0.768	0.841	0.878
Modularity	0.432	0.746	0.518	0.647
Average path length	2.543	3.396	2.246	2.161
